# Probing the physical nature and composition of signalsomes

**DOI:** 10.1186/1750-2187-6-1

**Published:** 2011-01-11

**Authors:** Hsien-yu Wang, Craig C Malbon

**Affiliations:** 1 Departments of Physiology & Biophysics, Health Sciences Center, School of Medicine, State University of New York at Stony Brook, Stony Brook, NY 11794-8661 USA; 2 Department of Pharmacology, Health Sciences Center, School of Medicine, State University of New York at Stony Brook, Stony Brook, NY 11794-8651 USA

## Abstract

**Background:**

Recent advances in our understanding of cell signaling have revealed assemblies of signaling components often viewed in fluorescence microscopy as very large, irregular "punctae". These punctae are often dynamic in nature, appearing to act as mobile scaffolds that function in integrating protein-protein interactions from large arrays of signaling components. The visualization of these punctae, termed "signalsomes" when applied to protein assemblies involved in cell signaling provokes the question, what is the physical nature of these structures made visible in live cells through the expression of fluorescently-tagged fusion molecules?

**Results:**

Steric-exclusion chromatography on wide-bore matrices, fluorescence correlation spectroscopy, and advanced proteomics permits the analysis of several important physical properties of signalsomes. Wnt canonical signaling is essential to normal cell development and dysregulation can lead to cancers in humans. Punctae/signalsomes have been reported based upon the study of fluorescently-tagged mammalian Dishevelleds. Dishevelleds are phosphoprotein scaffolds that demonstrate dynamic character and mobility in cells stimulated with Wnt3a. Recent studies have successfully isolated Dvl3-based signalsomes from mouse totipotent embryonic teratocarcinoma F9 cells in culture and sized by application of steric exclusion chromatography (SEC), displaying large discrete *M*_*r *_(0.5 and 2 MDa). Activation of the Wnt canonical β-catenin/LEF-Tcf-sensitive transcriptional response leads to an upfield shift of >5 MDa of the Dvl3-based signalsome. Fluorescence correlation spectroscopy (*fcs*) is a single molecule analysis performed in live cells that experimentally measures the diffusion coefficient and permits calculation of MW of the signalsome (0.2 and 30 MDa species in vivo), which also reveal an upfield shift in MW in response to Wnt3a. Proteomics provides for molecular dissection of the composition of the signalsome isolated from untreated and Wnt3a-treated cells.

**Conclusion:**

Dvl3-based punctae/signalsomes made visible by fluorescent microscopy now can be interrogated by advanced physical means, defining such properties as signalsome *M*_*r*_/MW, molecular composition, and intracellular locale.

## Background

Dsh/Dvls are unique phosphoprotein scaffolds essential for normal cell signaling and development in the animal kingdom[1-5]. Dvls organize and mediate Wnt signaling by the canonical (Wnt/β-catenin), non-canonical (Wnt/cGMP phosphodiesterase/Ca^2+^) and planar cell polarity (PCP) pathways[2,4,6-9]. How Dvl, a scaffold, with many dynamically interacting partners (e.g., Frizzle1[10], casein kinase 2[11], casein kinase1[12,13], Daam1 [14], etc.) operates is an overarching question in biology, no less in development and cell signaling. Our ability to visualize new paradigms is essential to developing new ideas in cell signaling. The Singer-Nicholson model of membrane protein interactions [15], for example, moved the field forward. Yet, to the extent that it became incompatible with what we have learned in the last 30 yr, that paradigm subsequently held us back conceptually. Kinetics of rapid signaling operated so fast that lateral diffusion of free-floating membrane proteins gave way to our envisioning solid-state signaling devices like ligand-gated ion channels and effectors that were assemblies of molecules of different character (e.g., receptors, G-proteins, effector paradigm). New strategies have yield greater protein-protein interactions (e.g., docking, assembly, undocking) with increasing complexity. Dvls highlight this evolution in thinking, being mobile, dynamic scaffolds that integrate interactions among protein kinases, phosphatases, adaptor molecules, receptors, and other scaffolds (e.g., LRP6/Axin complexes at the membrane)[9,16-19]. The Wnt homepage (http://www.stanford.edu/group/nusselab/cgi-bin/wnt/) provides an invaluable scoreboard, tallying new suspected Dishevelled-interacting proteins (DIPs). So, we might anticipate that for at least some of the time, Dvls operate as scaffolds that are *super*molecular complexes.

Florescence microscopy from several labs (including the authors') uniformly reports the presence of Dvls as "punctate" structures in cells (using immune-tagged and auto-fluorescent-tagged Dvls)[20-27]. Dr. Mariann Bienz's lab provided the first compelling images of Dvl2 that were shown to link to dynamic function of Dvl [22]. The existence of such punctate assemblies of GFP-tagged Dvls raises provocative questions about the physical properties of such assemblies *in vivo*, including: How large are the complexes?; Where are they localized intracellularly?; How many other DIP partners are found in Dvl-based supermolecular complexes?; and, How does the composition of these punctate structures change in response to Wnt stimulation? We now know much more about the nature of these punctae: they are large, are multi-protein (or supermolecular), are not lipid vesicle-associated (although may dwell at the membrane)[22], include all 3 Dvls, and their "appearance" is changed in response to Wnt stimulation of the cells. The hypothesis is that such visualized structures are supermolecular complexes with other DIPs involved critically in Wnt signaling.

All three Dvls microscopically appear as "punctae"[20,22,23,26,27], although the molar relationship among Dvl1, 2, and 3 is not that of equivalence. The abundance of each mammalian Dvl isoforms, in fact, is rather largely asymmetrical, i.e., Dvl2 constituting >90% of Dvl pool in a variety of mammalian cells [28]. Dvl1 and Dvl3 were found to be in far lower abundance, 2-5% each [28]. All three Dvls are necessary for Wnt signaling by the canonical and non-canonical pathways to function[23,28]. Yet, differential effects of siRNA-based suppression of each isoform are observed for Wnt signaling. Knock-down (KD) of Dvl3 has the most profound effect on Wnt/β-catenin signaling [28]. Knock-down of Dvl3 similarly differentially affects the function of the non-canonical pathways [23]. Likewise Dvl3-/- "knock-out" mice show the most profound changes in phenotype [29]. On this basis of KD studies of each isoform and differentially greater effects of overexpression of Dvl3 on activation of the Wnt canonical pathway that study of Dvl-based supermolecular complexes was performed in the context of Dvl3. Dvl3 displays DIX, PDZ, and DEP domains, highly conserved in mammalian Dvls[8,30-32]. More than 25 DIPs are suspected to dock to each of the Dvls; nearly all DIPs are thought to dock at the N-terminal 2/3^rd ^of the Dvl[9,33]. The C-terminal 1/3^rd ^(C-terminal to the DEP domain) is largely bereft on known DIPs[9]. This C-terminal 1/3^rd ^of Dvl3 is the region of highest non-identity among Dvls and remains a region deserving of detailed interrogation for high-value DIPs, such as K-homology splicing regulator protein (KSRP)[34] and Ras GTPase-activating protein-binding protein 1 (G3BP1, unpublished results), which function in Wnt regulation of RNA.

How are such punctae to be interrogated, their physical nature and composition established? The punctate structures revealed for Dvls by fluorescence microscopy in live cells are suggestive of supermolecular complexes of large size. Biochemical analysis of individual Dvls by sodium dodecyl sulfate-polyacrylamide gel mobility has revealed that they form Dvl-based oligomers [20,22]. Homodimeric Dvl2 and Dvl3 have been characterized [20,35]; the binding is tight and dissolution to monomers requires use of a strong chaotropic agent (8 M urea)[36]. Pull downs of each Dvl isoform reveal the presence of all three Dvls [23,28]. The relative size of such signaling complexes remained enigmatic and difficult to address by conventional means. One can ask if it legitimate to even refer to these supermolecular complexes as Dvl-based? The answer is "yes", as Dvl3 expression regulates the formation of the punctae. Knock-down of Dvl3 expression provokes a sharp reduction in punctae formation by remaining Dvls [36]. Taking existing studies into consideration, a novel hybrid strategy to interrogate Dvl3-based complexes emerged that was based in three approaches: isolation of the complexes from cell lysates in tandem with steric-exclusion chromatography (SEC); fluorescence correlation spectroscopy (*fcs*) in live cells; and, proteomic interrogation of Dvl3-based punctae, assuming that such punctae would survive biochemical isolation by SEC.

## Results

### Isolation and sizing of Dvl3-based signalsomes by steric-exclusion chromatography (SEC)

**Figure 1:**
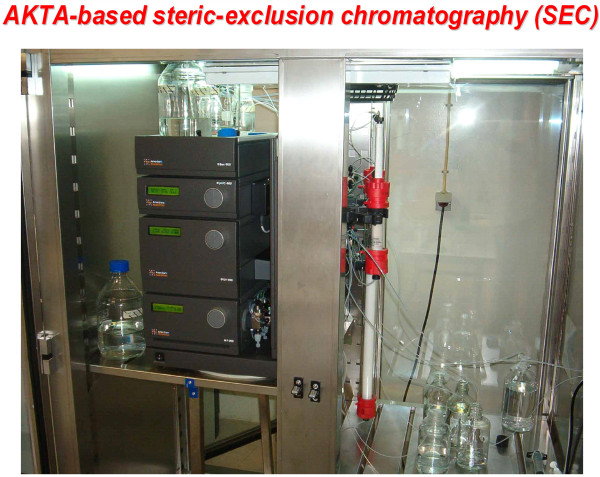
**Steric-exclusion chromatography enables isolation and molecular sieving of large, supermolecular complexes/signalsomes**. One configuration of steric-exclusion chromatography optimized in the authors' laboratory makes use of the Pharmacia AKTA-based liquid handler equipped with large diameter, long (30-60 cm) columns packed with wide-bore matrices such as Sephacryl 200, 400, or larger. The system can accommodate large sample loading and provides excellent performance in efforts designed to isolated very large supermolecular complexes like the Dishevelled3-based signalsomes highlighted in this article.

Based upon the size of other large supermolecular devices in cells (e.g., protein synthetic complexes [37,38], nuclear pore complex [39]), we adopted two complementary approaches [36]. The first, SEC, is capable of isolating and sizing (*M*_*r*_) the complexes as well as enabling subsequent proteomic analysis, although requiring significant amounts of starting material. The second operates at the cell level using a single (or scanning) 1.5 femtoliter voxel and conditions in which expression of an autofluorescently-tagged molecule are maintained very low (5-50 molecules/cell). Under such conditions, *fcs *offers unparalleled analysis of calculated mass (MW) of complexes based in autofluorescently-tagged Dvl3. Isolating such "super" molecular complexes offers new challenges that render most common SEC techniques irrelevant, incapable of sieving such large molecular structures. SEC matrices more commonly found in industrial settings were exploited for these analyses. Very long column (60-90 cm), commercially packed under stringent conditions, can be adapted to provide optimal separation of large supermolecular complexes (Figure 1). Using this approach, Dvl3-based complexes, with sizes ranging from 0.2 to >5 MegaDa (MDa)-*M*_*r *_became addressable for physical separation by steric-exclusion chromatography [36]. When isolated from mouse totipotent teratocarcinoma F9 cells, Dvl3-based supermolecular complex display two dominant species with *M*_*r *_~0.2 and 0.8-2.0 MDa. When Dvl3-based supermolecular complexes are isolated from F9 cells treated with Wnt3a for 30 min, the *M*_*r *_of the dominant peak displays an upfield shift of >5 MDa, derived at the expense of the lower-*M*_*r *_peak. The assembly of these large complexes occurs in advance of the downstream activation of the Lef/Tcf-sensitive transcription by Wnt3a. Study of Wnt canonical pathway activators versus inhibitors of signaling agree well with the ability of these treatments to stimulate versus block the assembly of Dvl3-based supermolecular complexes.

### Analysis of MW of signalsomes *in vivo/in situ *by fluorescent correlation spectroscopy (*fcs*)

**Figure 2:**
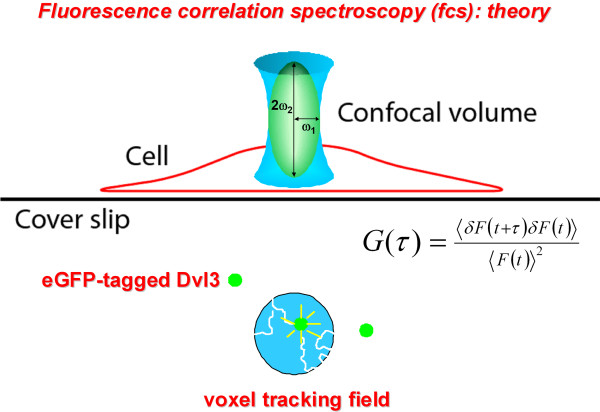
**Fluorescence correlation spectroscopy enables measurements of diffusion constants and calculate MW of fluorescently-tagged molecules core to supermolecular complexes of interest, such as the Dvl3-based signalsomes**. *Fcs *is a single molecule analysis of the dwell time of a molecule/complex of interest, sampled in situ in live cells. Please consult the Methods section for a detailed explanation of the theory and practical aspects of applying *fcs *measurements in live cells.

The second technology exploited was fluorescence correlation spectroscopy, *fcs*, an extremely powerful single-molecule technique for deducing MW of fluorescently-labeled targets from measured dissociation constant, *D*, in live cells (Figure 2). The dwell time of a single fluorescently-labeled fusion protein (e.g., eGFP-Dvl3) is tracked within the limits of a very small voxel. As described in the *Methods *section, data from hundreds of samplings from different cells measured in the cytosol can yield a diffusion coefficient from which the MW of the object (in this case a supermolecular complex) can be calculated with precision. After several years of testing instruments and capabilities elsewhere, an *fcs *core facility was created, featuring two high-grade *fcs*-equipped fluorescence instruments. For some technologies, investigator-based training and experience is sufficient for most research applications. Based upon our own experience, an *fcs *center requires close collaboration with biophysicists who use these instruments in their own research to provide advice and assistance in applying *fcs *to new problems in cell signaling. The results of several years of experience have been both unexpected and remarkably exciting. We succeeded in performing *fcs *of eGFP-tagged Dvl3 in F9 cells as well as HEK293 cells[36]. The mass of Dvl3-based complexes established by *fcs *of cells absent stimulation by Wnt3a is very large, observed in discrete sizes that display MW ranging from 25-35 MDa. Sodium dodecyl sulfate-polyacrylamide gel electrophoresis (SDS-PAGE), SEC (molecular dissection of isolated peaks), and *fcs *(biophysically monitored in live cells *in situ*) uniformly detect Dvl monomers and the dimers with high precision and good agreement. Significantly, establishing the physical nature of the large (05.-5 MDa, addressable by SEC) and very large (25-35 MDa, addressable by *fcs*) Dvl3-based complexes brings us back to our initial goal of interrogating large punctae of Dvls observed in cells undergoing activation with Wnt.

Are these large/very large supermolecular complexes simply large polymers of Dvls? By no means. We have been able to use the SEC coupled to proteomics to identify the DIP constituents of the large Dvl3-based complexes. As others have reported [22], we find no evidence that such "punctate" complexes are vesicle-bound, i.e., no presence of sufficient lipid or a buoyant density to establish them as vesicle-bound *per se*. Is it possible to interrogate the nature of the proteins found in the Dvl3-based complexes? Initially this seemed impractical, since the yield of supermolecular complexes through a standard preparation was limiting. By migrating to mega-preps derived from 2-3 liters cell cultures of F9 cells, molecular dissection of Dvl3-based complexes isolated as distinct peaks from SEC by modern proteomic strategies became a reality.

As noted, SEC analysis successfully identified Dvl3-based complexes of large MW that displayed a >5 MDa-upfield shift in mass in response to Wnt3a stimulation[36]. Yet the *fcs *analysis revealed complexes in untreated F9 cells with MWs greater than those detected by SEC[36]. The *fcs *readily identified smaller monomeric and oligomeric assemblies of Dvl3, established by SEC and SDS-PAGE alike [36], providing good agreement and calibration at the low end of the analyses. The hypothesis being tested is that the larger MW of the complexes identified by *fcs *reflects true higher-order complex structure (e.g., docking, oligomerization, mean dwell time of transient membrane association). Many non-covalent associations, however, are labile to shear forces that can be generated in SEC. Significant differences in apparent masses can be anticipated between *in situ *between *fcs *and SEC analyses. Further testing of industrial, large-pore matrices likely will continue to extend the upper reach of SEC from 10 to 40 MDa-*M*_*r*_.

What about *fcs*, what distinct advantages does it afford us? An initial worry with *fcs *might be that its sensitivity and precision might be too limited. The SEC and *fcs *analyses of Dvl3-based complexes evolved in tandem and it became clear fortunately that the size of the Dvl3-based supermolecular complexes were ideal for *fcs *determination. The strengths of *fcs *are that one can obtain calculated MW of eGFP-tagged Dvl3-based supermolecular complexes in live cells, under native conditions, sampling intracellular domains through precision placement of the very small voxel in which the targets are tracked (Figure 2 and *Methods *section).

### Proteomic analysis of the composition of isolated signalsomes

The third strategy necessary to address the initial hypothesis on the composition of the Dishevelled-based punctae is proteomics. Proteomic analysis offers an ideal strategy with which to interrogate the composition of Dvl3-based supermolecular complexes. Assuming sufficient starting materials from peaks of Dvl-based complexes isolated by SEC, the goal would be to interrogate both the peaks isolated from untreated cells as well as those upfield, higher-*M*_*r *_peaks observed only when cells were stimulated with Wnt3a. One cardinal concern is always that of the potential presence of contaminating proteins/complexes of *M*_*r *_similar to those that are Dvl3-based. The upfield shift in apparent mass observed in the Dvl3-based complexes isolated from Wnt3a-treated cells holds the key to this challenge. Operationally, unrelated proteins/complexes "contaminating"(i.e., not related to Wnt signaling) Dvl3-based supermolecular complexes isolated from untreated cells by definition would not co-migrate with the higher *M*_*r *_Dvl3-based assemblies formed in response to Wnt3a. The upfield shift of >5 MDa moves the Dvl3-based complexes to a less populated region of the chromatogram. SEC and proteomics employed in tandem have born this out in early experiments, i.e., DIPs and some new classes of signaling proteins have been identified. Within this novel group, proteins involved in mRNA stability and AKAPs (AKAP5 and AKAP12) have emerged (unpublished data) and are of keen interest. The ability to contrast the proteomic profiles of Dvl3-based peaks isolated from untreated versus Wnt3a-treated cells thus becomes an enabler for focusing upon DIPs in the Dvl3-based complexes. Proteomic identification of complex constituents will enable determination of docked/undocked partners during Wnt activation.

**Figure 3:**
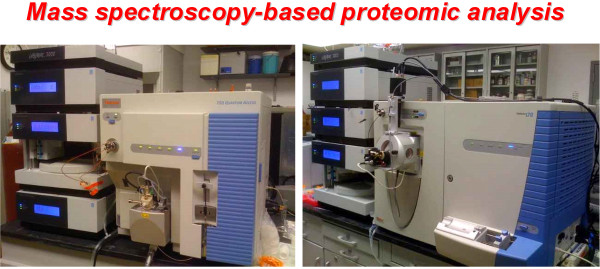
**Mass spectrometry-based proteomic analysis enables identification of the proteins that constitute large supermolecular complexes such as Dvl3-based signalsomes**. The ability to fragment and subsequently sequence a complex of proteins, such as the Dvl3-based signalsome, is essential for similar efforts aimed at establishing the identity of protein partners involved in large complexes that integrate and propagate signaling downstream. These mass spectrometry-based analyses can be performed in parallel or sequentially to optimize the discovery of constituent peptides.

Dvl3-based supermolecular complexes subjected to such molecular dissection yield much new information. A typical mega-prep of cells yields about 0.5-0.7 mg of protein in the 2-5 MDa peaks. Ten to 20% of the pooled peak sample is consumed for each analysis subjected to fragmentation and to liquid chromatography electrospray ionization tandem mass spectrometry (LQ-ESI-MS-MS) analysis (Figure 3). Isolating peak samples from large cultures subjected to SEC clearly is labor and cost-intensive, but invaluable. This strategy is to first isolate SEC-sized discrete peaks of Dvl-based complexes, focusing upon peaks with signature retention times/volumes (e.g., 0.5, 2.0 and 5-7 MDa-*M*_*r *_peaks). If "contamination" of the lower *M*_*r *_peaks is suspected, peaks can be pooled and subjected to field IEF in porous gels, to characterize, enrich, and identify (if possible, by IEF precipitation) Dvl3-based complexes, segregated from other "contaminating" complexes. Such highly-enriched peaks can be subjected to proteolytic degradation and proteomic analysis by LQ-ESI-MS-MS, requiring a state-of-the-art proteomic center with staff experienced in probing low abundance targets in complex mixtures. Key molecules of known function have been readily identified in Dvl3-based 2 MDa-*M*_*r *_peaks, assembled in response to Wnt3a and activation of the Wnt/β-catenin canonical pathway (e.g., all three Dvl isoforms, Axin, Axin2, CK2, CK1α and γ, and GSK3β). Novel molecules, such as AKAP5 and AKAP12 that are key in cAMP-based signaling, were newly identified (unpublished observations). Positive identification is ascribed only to a protein for which at least 10 unique fragments have been fully sequenced, from multiple runs, and multiple samplings. What can be accomplished through such a marriage of SEC and proteomics is really impressive: identifying large order assemblies, discerning constituent partners and following dynamic assembly in response to signaling pathway manipulation.

## Discussion

The ability to create fusion proteins of interest with fluorescent moieties created the ability to interrogate these proteins dynamically in live cells. Consistent with earlier immunohistochemical and immunofluorescence analysis, autofluorescently-tagged signaling proteins (e.g., receptor, G-proteins, effectors) were often observed as "puncta". These puncta were large, irregular in shape, and the subject of speculation as to their actual mass and composition. For heterotrimeric G-protein-based signaling, punctate structures were the rule, not the exception.

Wnt signaling is heterotrimeric G-protein-linked in flies as it is frogs and zebrafish. Downstream of intrinsic membrane proteins, e.g., Frizzled-1 and LRP6, involved in canonical Wnt signaling are structures which integrate and propagate the upstream signals. Dishevelleds are core proteins in virtually all aspects of Wnt signaling and were shown early to form large irregular punctae by a variety of optical based analyses. Known to dock important protein kinases, adaptors, and phosphatases, Dvls provided an ideal test case in which to ask more detailed queries about the size, composition, and dynamic character of these phosphoprotein scaffolds, characterized earlier as simply punctate structures.

As recently reported, Dvl-based punctae have been shown in fact to be large supermolecular structures that can be prepared from cells and sized by steric exclusion chromatography. The Dvl3-based structures can be subjected to molecular sieving on wide-bore matrices packed in long columns calibrated and operated with state-of-the-art FPLC systems. The peaks identified are discrete, displaying highly reproducible *M*_*r*_. This approach demonstrated that Dvl-based supermolecular complexes could be prepared and their hydrodynamic properties characterized by SEC. This advance demonstrated feasibility, which was the overarching challenge to establishing what these punctate signalsomes are physically.

It was essential to gain insights into earlier observations that the Dvl-based punctae are influenced by treating cells with Wnt3a, activating the canonical beta-catenin, Lef/Tcf-sensitive transcriptional response. Interrogation of the Dvl3-based complexes by SEC revealed a marked upfield shift in *M*_*r *_of >5 MDa. Furthermore, activation of the canonical pathway in the absence of Wnt3a which stimulates Lef/Tcf-sensitive transcription was preceded by formation of the Dvl3-based signalsomes. Blocking the ability of Wnt3a to activate the canonical pathway at several distinct levels likewise blocked signalsome formation. Thus the linkage between signalsome formation and function was established in this pathway.

Physical isolation of the Dvl3-based signalsomes was an advance, but would the composition of resolved peaks of the SEC-sized supermolecular complexes be addressable? As noted above, these peaks migrated to upfield of much of the proteome/complex profile. Even at *M*_*r *_of 1-2 MDa of the chromatogram, the amounts of total protein present in that region of the profile has fallen off dramatically from that observed at 0.2-0.5 MDa (unpublished observations). In addition, the sharp large (>5 MDa) upfield shift provoked by Wnt3a alone would enrich the Dvl3-based signalsomes, moving these signalsome well away from "contaminating" complexes encountered downfield. Starting with mega-preps of cells in culture, sufficient material can be prepared to perform proteomic interrogation of peaks of supermolecular complexes like the Dvl3-based complexes, to find out something about these very large punctae/signalsomes. Constituent partners that might be expected (e.g., Dvl1, Dvl2, Dvl3, Axin) as well as unexpected, curious partners (e.g., A-kinase anchoring proteins, AKAPs) were identified by LQ-ESI-MS-MS. The application of field isoelectric focusing (FIEF) of the signalsomes in large-pore gels matrices may provide an additional enrichment. By comparing the proteomic profiles from the 1 MDa-*M*_*r *_complexes (absence of Wnt stimulation) versus >5 MDa-*M*_*r *_complexes (assembled in response to Wnt3a), it will be possible to define the relative abundance of partners, assuming that the ionization of the fragments is equivalent. Through such a strategy other supermolecular complexes/signalsomes assembled in response to signaling become fully addressable. For any such supermolecular complex to assemble in a bona fide signalsome, the assembly must occur in advance of the propagation of the signal downstream. For Wnt canonical signaling, the assembly of the upfield Dvl3-based signaling complexes is largely completed by 30-60 min, well before the Lef/Tcf-sensitive transcriptional activation occurs.

The *fcs *capabilities offer many novel and powerful insights for the study of signalsomes. As a single molecule analysis that can be targeted for sampling in discrete locales in live cells, *fcs *is only beginning to be exploited for the study of signalsome mass and dynamic mobility. The Dvls, shown to display mobility in cells stimulated with Wnt3a, offer an ideal test case for sampling the diffusion coefficient of their signalsomes in perinuclear, cytosolic, and juxtamembrane regions of the cell. The hypothesis to be tested is that the apparent MW of these Dvl-based signalsomes will reflect changes in size as the signalsome is assembled and also where in the cell the assembly is occurring. Upon docking to the inner leaflet of the membrane, for example, the apparent MW would increase infinitely when the diffusion coefficient is derived from a molecule essentially immobilized at the membrane. But this scenario remains largely speculation, awaiting empirical analysis in cells undergoing activation of the Wnt canonical pathway using autofluorescently-tagged Dvl3 (or other tagged key signaling molecules) as the signal.

The differences between the apparent *M*_*r *_derived from SEC and the MW calculated by *fcs *for the Dvl3-based signalsomes of unstimulated cells deserve some interrogation. The MW of the Dvl3-based supermolecular complex is several-fold larger than that reported on the bases of SEC analysis. The MW determined by the *fcs *data benefits from the fact that this single molecule analysis is performed *in situ*, in live cells. This is not to say that *fcs *estimates of MW are without bias, since the strategy does require assumptions about the hydrodynamic properties of the complex, such as axial ratio and partial specific volume, that potentially bias the calculations. On the other hand, the SEC analysis provides a very precise *M*_*r *_for complexes subjected to molecular sieving. Thus, it must be kept in mind that the shear force created as a result of the size of the complex, the pressure under which the molecular sieving is being performed, and the viscosity of the fluid can be considerable. It is possible that the large and very large complexes under study may have some higher order structure of assembly that is lost to the shear force of SEC. Attempts either to minimize the shear force by reducing the SEC pressure to the point of just overcoming back diffusion or to change the matrix employed for SEC to an ever larger bore may provide an avenue by which to interrogate the role of shear force, if any, in jeopardizing the integrity of a much larger Dvl3-based complex. Alternatively, sampling *fcs *from numerous subcellular locales, e.g., cytosol, perinuclear, and juxtamembrane, may reveal Dvl3-based complexes in live cells with MW in closer agreement with those assigned by SEC.

Researchers who study cell signaling do so from a broad array of disciplines and interests. For development and Wnt signaling in particular, the approaches are diverse, providing a collect synergy that has brought major advances to the study of early development. Dishevelled is a phosphoprotein scaffold that has a critical, but not fully understood, role in development, examined in the fly, frog, zebrafish, mouse and human species. Dishevelled has been under interrogation by microscopy for years, studies uniformly revealing this key signaling protein as dynamic "punctate" structures of relatively large dimensions. But just what in fact are such large signaling punctae observed in the context of cell signaling? A fundamental hypothesis from the microscopic analysis to the current day can be stated simply in the question, "what is the physical nature and functional significance of signaling punctae, often referred to as signalsomes?" Three strategies are briefly described herein that afford unprecendented ability to address the central question. Assuming one can break open the cell and retain the punctae, it is possible to subject these complexes to SEC to arrive at an approximation of the *M*_*r *_of the large, supermolecular entities such as signalsomes. Testing the function of such complexes, if addressable once isolated from the cell, is rather challenging, even when the system of Wnt signaling is involved. There are numerous tools that can be applied to the Wnt canonical pathway to either stimulate or inhibit signaling at various levels. Used judiciously, these tools can probe the extent to which complex and formation and function are related. For Dvl3-based signalsomes the structure/function relationship seems quite strong. Key mutants of Dvl3 have been identified that block signalsome formation in response to Wnt3a. These have been shown to block downstream signaling and can be understood in the context of interrupting important protein-protein interactions of Dvl. Ideally, one would seek to identify mutations in either Dvl or Axin that interfere with assembly of Dvl3-based signalsomes, although scanning for such mutants randomly would be laborious. Structure-function studies of Dvl3 (or other molecules like Axin key to Wnt signaling) can yield insights, directing one where to look first for interesting protein-protein interactions essential to the formation of complex/signalsomes and downstream signaling. Higher-order assemblies, identified by *fcs*, may be based upon weak interactions that are susceptible to the shear force (or cell dissolution) generated by the SEC under current conditions.

The *fcs *data benefits from its ability to be extracted from rather small volumes in discrete regions of the cells *in vivo*. This benefit is offset, to some extent, by our ignorance of other cell constituents that may be *bona fide *docking sites for signalsomes. Even transient docking of signalsomes to other cellular entities can artificially increase the MW derived from *fcs*, especially for proteins with significant dwell times in docking. Docking to the inner leaflet of the cell membrane to LRP6 or Frizzled GPCRs would reduce the diffusion coefficient of the signalsome, which would artificially increase the apparent MW in the final analysis by *fcs*. Modern proteomic capabilities provide a portal to identify complex partners assembled into signalsomes. When activation of a pathway leads to a physically larger signalsome (as shown for Wnt3a stimulation of Dvl3-based signalsome), samples derived from basal as opposed to stimulated cells can be subjected to proteomics to address what partners have been added/lost to the signalsome as it assembles to propagate the signal downstream? On the surface, the intersection of SEC, *fcs*, and proteomics would seem unbeatable in sorting out the structure/function of very large supermolecular complexes encountered in a variety of cell signaling pathway. Whether or not such cautious optimism will proves well-founded remains to be seen. Painstaking application of this powerful trio of techniques to a broader array of targets, not just signalsomes, will undoubtedly be required to test the central hypothesis. The Dvl3-based signalsome has taken on a decidedly new look based upon recent advances in technology; this outcome alone was worth the investment of time and resources.

## Conclusions

Steric-exclusion chromatography, fluorescence correlation spectroscopy, and advanced proteomics afford biologists powerful new ways to interrogate structure/function relationships of very large supermolecular complexes, typified by Dishevelled-based signalsomes involved in Wnt signaling in early development.

## Methods

### Cell culture and transfection

Mouse F9 teratocarcinoma cells were obtained from the ATCC collection (Manassas, VA). Cells were cultured in Dulbecco's modified Eagle's medium (Cellgro, Manassas, VA) supplemented with 15% heat-inactivated fetal bovine serum (Hyclone, South Logan, UT) at 37°C in a humidified atmosphere with 5%CO_2_. For transfection, plasmid DNA was introduced into cells by using LipofectAMINE and PLUS Reagent (Invitrogen) as per the manufacturer's instruction. Stable clones were selected in medium containing G418 (0.4 mg/ml).

### Construction of plasmids

Expression vector pcDNA3.1 harboring eGFP-tagged human Dvls (hDvl1-eGFP, or hDvl2-eGFP or hDvl3-eGFP) was constructed as described [23]. Human Dvl3 and Dvl3-CT carrying point mutations were generated by using QuickChange Site-Directed Mutagenesis Kit (Stratagene), following the manufacturer's instructions. The authenticity of all constructs was confirmed by direct DNA sequencing.

### Fluorescence correlation spectroscopy

The *fcs *measurements were performed in the Fluorescence Correlation Spectroscopy Center at Stony Brook (Department of Physiology & Biophysics) using a Zeiss LSM 510 Meta/Confocor 2 apparatus (Jena, Germany) fitted with a 40X NA 1.2 C-Apochromat water-immersion objective.

Standard configurations and minimal laser powers are employed to avoid photobleaching. Routinely, the pinholes are adjusted at least daily and the detection volume calibrated by measuring the diffusion of Rhodamine (Rh6G, *D *= 3 × 10^-6 ^cm^2^/second) in water [40]. All measurements are conducted at room temperature. The eGFP moiety is excited with the 488 nm line of argon ion laser and collected emission spectra through a 505 LP filter. Software provided by Zeiss was employed to fit the autocorrelation curves to the model equation for free Brownian diffusion typically employed for *fcs *[41]:

$$G(\tau )=\frac{1}{N}\cdot \sum_{i}\frac{Y_{i}}{1+\frac{\tau }{\tau_{d,i}}}$$

where N is number of molecules in the detection volume, Y_i _is a fraction of molecules diffusing with diffusion coefficient *D*_i _producing residence times τ_d,i_. We calculated the diffusion coefficient, *D*, from the Einstein relation:

$$D=\frac{r^{2}}{4\tau_{d}}$$

To estimate the approximate molecular weight (MW) of diffusing complexes the complexes are assumed to be spherical and the data processed using the following equation to determine the hydrodynamic radius:

$$D=\frac{kT}{6\pi \eta R}$$

where, k is Boltzmann's constant, T is the temperature, η is the viscosity of the solvent, and R is the hydrodynamic radius. MW is related to R by following equation:

$$V=MW\bar{\nu }=\frac{4}{3}\pi R^{3}$$

where, V is volume and $\bar{\nu }$ is specific gravity. To account for the viscosity of the cells we used values obtained for GFP in cells, D = 1.7 × 10^-7 ^cm^2^/s and the MW = 27 kDa.

### Proteomics

The liquid chromatography electrospray ionization tandem mass spectrometry (LQ-ESI-MS-MS) analyses were performed at the Stony Brook Proteomic Center at the Health Science Center (http://www.osa.sunysb.edu/Proteomics/Instrumentation.html, accessed 12062010).

### Steric-exclusion chromatography

Samples were first filtered (0.45 μm) and diluted with buffer. Typically 20 mg protein is applied to the Superdex 200 (or 400) gel filtration column (HiLoad SuperdexTM 200 prep grade 26/60, fast-performance liquid chromatography system AKTA, GE Healthcare), pre-equilibrated with 20 mM Tris-HCl, pH 8.0, 0.2 M NaCl, and 1.0% glycerol. The fractions collected were maximally 0.9 ml/each. Each fraction was analyzed by SDS-PAGE and immunoblotting. Protein concentration was determined using the Bradford assay. For more precise analysis of 1- 7+ MDa complexes, cell lysates were subjected to SEC on a Sephacryl S-400 gel filtration column (HiPrep Sephacryl S-400 high-resolution column) operated by fast-performance AKTA liquid chromatography (GE Healthcare).

## Abbreviations

DMEM: Dulbecco's modified Eagle's medium; Dvl1: Dishevelled-1; Dvl2: Dishevelled-2; Dvl3: Dishevelled-3; Dsh: Fly Dishevelled; F9: mouse totipotent embryonal teratocarcinoma F9 cells; HEK 293: human embryonic kidney 293 cells; *fcs*: fluorescence correlation spectroscopy; KD: knock-down; KSRP: K-homology splicing regulator protein; LQ-ESI-MS-MS: liquid chromatography electrospray ionization tandem mass spectrometry; Rfz1: rat Frizzled-1; SDS-PAGE: sodium dodecyl sulfate-polyacrylamide gel electrophoresis; SEC: steric-exclusion chromatography

## Competing interests

The authors declare that they have no competing interests.

## Authors' contributions

The authors contributed equally to the evolution of the study design; the authors contributed equally, performed the studies, gathered the data, and outlined a draft of the manuscript. HYW wrote the manuscript; CCM edited the drafts, read and approved the final version of this unpublished work. Each author read and approved the final manuscript.
